# Supplementary material for: Under the Cover of Darkness: A Transcriptomic Exploration of Clubroot During the Night
Source: Plant Direct. 2026 Jun 8;10(6):e70176. doi: 10.1002/pld3.70176 (PMC13247141; doi:10.1002/pld3.70176)
Supplement: Supplementary file 2 — Additional File 1: Detailed overview of the samples sequenced in this study. Additional File 2: PCA on control A. thaliana plants, showing the decreased influence of the temporal factor (i.e., day and night) on the overall gene expression levels with the aging of the plants. Additional File 3: Volcano plot showing the number of DEGs in the day (top figure) and night (bottom figure) in infected A. thaliana plants 14 DAI. Additional File 4: Volcano plot showing the number of DEGs in the day (top figure) and night (bottom figure) in infected A. thaliana plants 21 DAI. Additional File 5: All GO categories enriched in DEGs in A. thaliana roots 14 DAI infected by P. brassicae as in Figure 3. GO categories are organized by decreasing ‐log10 adjusted p‐value (i.e., top to bottom in the relative figure). Additional File 6: All GO categories enriched in DEGs in A. thaliana roots 21 DAI infected by P. brassicae as in Figure 3. GO categories are organized by decreasing ‐log10 adjusted p‐value (i.e., top to bottom in the relative figure). Additional File 7: GO terms overrepresentation analysis of clusters of DEGs driven by the interaction between infection and time of the Day 14 DAI. Additional File 8: Heatmap highlighting DEGs explained by the interaction between infection and time of the day as detected in infected A. thaliana roots 21 DAI. The row‐normalized and log transformed gene counts are shown for each sample separately. The status of each sample is described by a combination of green (control plant) or purple (infected plant) and yellow (day) or (blue) bars. DEGs with similar patterns of expression have been grouped in five clusters (numbers in white boxes). GO terms overrepresentation analysis of clusters (2, 3, and 4) of DEGs driven by the interaction between infection and time of the Day 21 DAI. Additional File 9: Enriched GO terms and corresponding genes underpinning Figures 2 and 3 before data reduction. [file PLD3-10-e70176-s002.docx]

## Supporting information

Additional File 1 Detailed overview of the samples sequenced in this study

| Condition | Age (days after infection) | Hours after artificial dawn | Replicate code | Raw reads |
| --- | --- | --- | --- | --- |
| Control | 14 | 5 hr 50' (day) | 127925 | 41193025 |
|  |  | 5 hr 50' (day) | 127935 | 50545664 |
|  |  | 5 hr 50' (day) | 127936 | 35205326 |
|  |  | 10 hr 40' (day) | 127906 | 56725609 |
|  |  | 10 hr 40' (day) | 127912 | 54273366 |
|  |  | 10 hr 40' (day) | 127918 | 38720139 |
|  |  | 15 hr 30' (night) | 127926 | 41309013 |
|  |  | 15 hr 30' (night) | 127931 | 55027843 |
|  |  | 15 hr 30' (night) | 127937 | 38004169 |
|  |  | 19 hr 20' (night) | 127907 | 39958547 |
|  |  | 19 hr 20' (night) | 127913 | 37101449 |
|  |  | 19 hr 20' (night) | 127919 | 40693049 |
| Infected | 14 | 5 hr 50' (day) | 127964 | 48508205 |
|  |  | 5 hr 50' (day) | 127970 | 41810661 |
|  |  | 5 hr 50' (day) | 127976 | 54739923 |
|  |  | 10 hr 40' (day) | 127946 | 42594195 |
|  |  | 10 hr 40' (day) | 127952 | 41155738 |
|  |  | 10 hr 40' (day) | 127958 | 58101982 |
|  |  | 15 hr 30' (night) | 127965 | 43247574 |
|  |  | 15 hr 30' (night) | 127971 | 46509846 |
|  |  | 15 hr 30' (night) | 127977 | 57602784 |
|  |  | 19 hr 20' (night) | 127947 | 40263406 |
|  |  | 19 hr 20' (night) | 127953 | 41398461 |
|  |  | 19 hr 20' (night) | 127959 | 50627500 |
| Control | 21 | 5 hr 50' (day) | 127908 | 52533389 |
|  |  | 5 hr 50' (day) | 127914 | 36118824 |
|  |  | 5 hr 50' (day) | 127941 | 64871718 |
|  |  | 10 hr 40' (day) | 127928 | 41179331 |
|  |  | 10 hr 40' (day) | 127933 | 45662748 |
|  |  | 10 hr 40' (day) | 127939 | 54002775 |
|  |  | 15 hr 30' (night) | 127909 | 43278660 |
|  |  | 15 hr 30' (night) | 127915 | 43201375 |
|  |  | 15 hr 30' (night) | 127920 | 42466381 |
|  |  | 19 hr 20' (night) | 127929 | 43287455 |
|  |  | 19 hr 20' (night) | 127934 | 46270564 |
|  |  | 19 hr 20' (night) | 127940 | 53657896 |
| Infected | 21 | 5 hr 50' (day) | 127948 | 59286277 |
|  |  | 5 hr 50' (day) | 127954 | 62740880 |
|  |  | 5 hr 50' (day) | 127960 | 46195940 |
|  |  | 10 hr 40' (day) | 127967 | 48074903 |
|  |  | 10 hr 40' (day) | 127973 | 61491275 |
|  |  | 10 hr 40' (day) | 127979 | 62580301 |
|  |  | 15 hr 30' (night) | 127949 | 55537283 |
|  |  | 15 hr 30' (night) | 127955 | 65514599 |
|  |  | 15 hr 30' (night) | 127961 | 54889653 |
|  |  | 19 hr 20' (night) | 127968 | 85469436 |
|  |  | 19 hr 20' (night) | 127974 | 71731948 |
|  |  | 19 hr 20' (night) | 127980 | 58536488 |

**
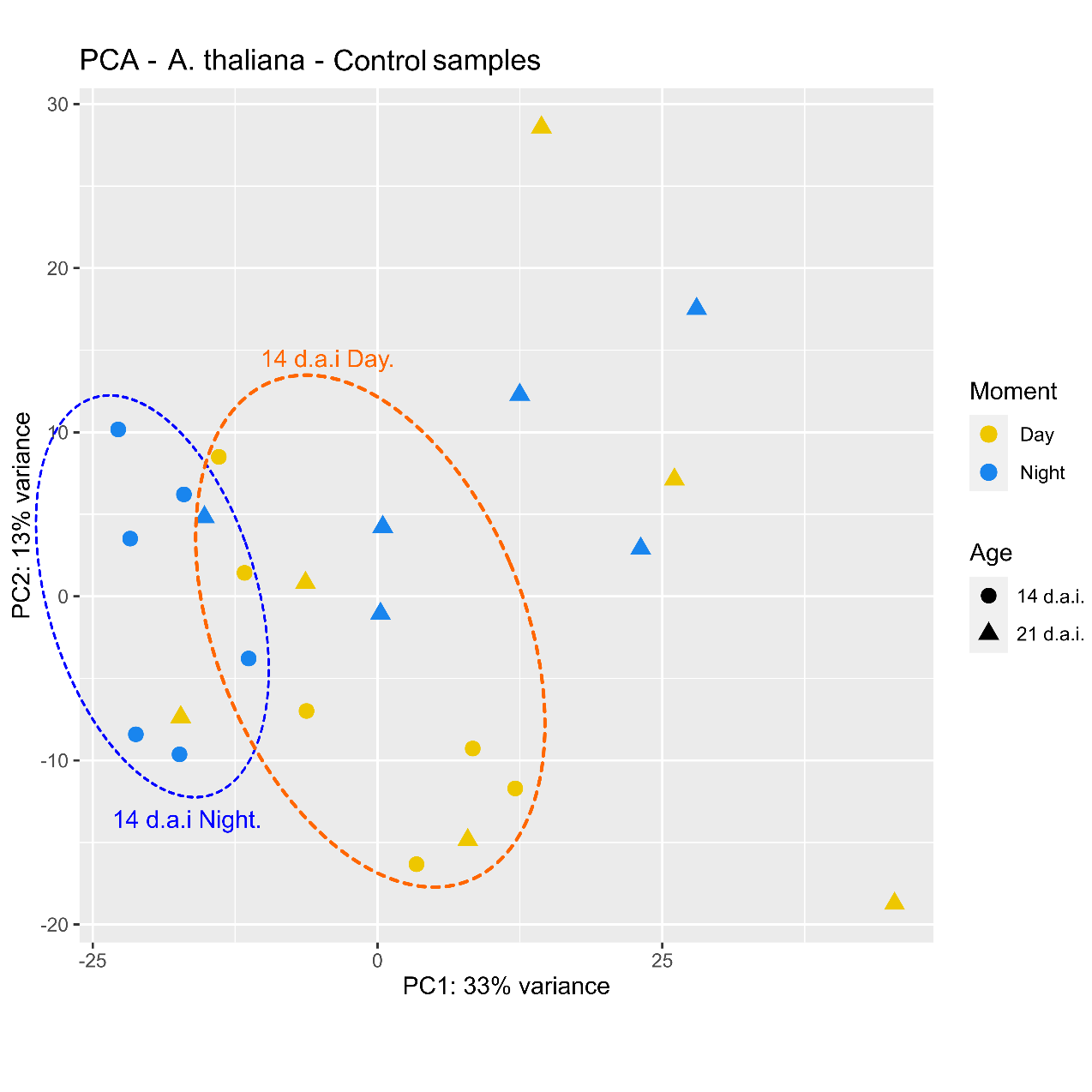
**

Additional File 2 PCA on control A. thaliana plants, showing the decreased influence of the temporal factor (i.e., day and night) on the overall gene expression levels with the ageing of the plants.

**
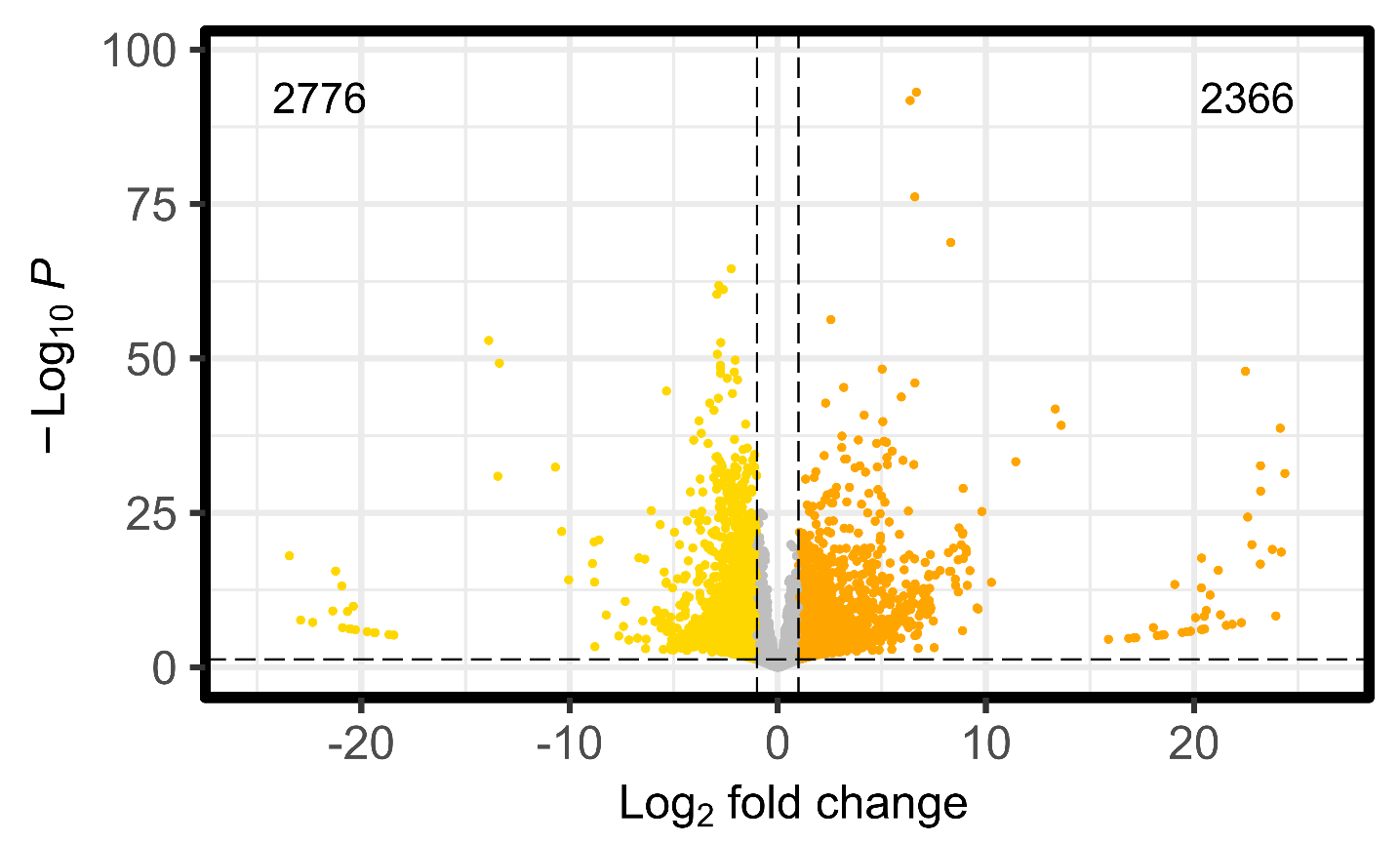

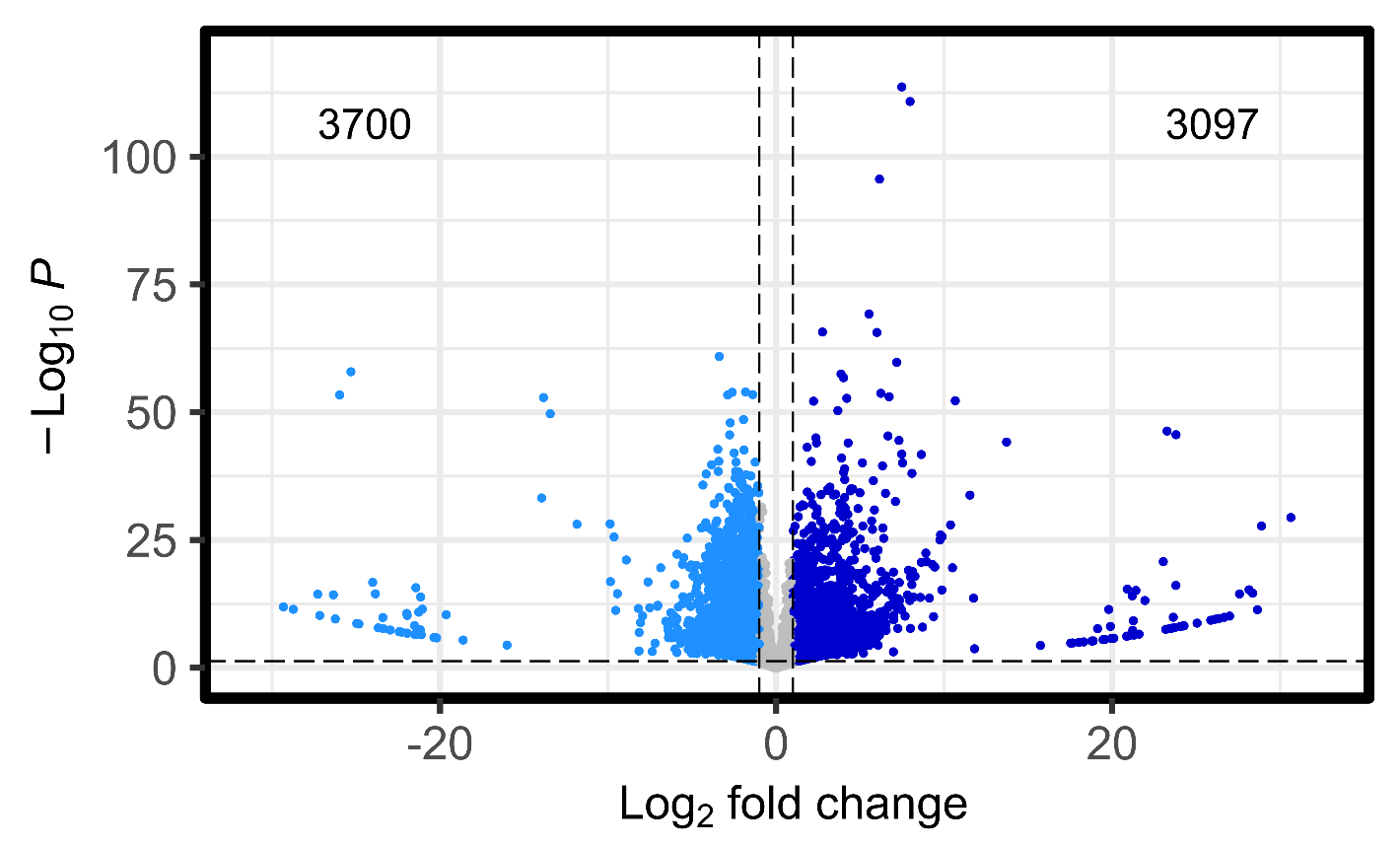
**

Additional File 3 Volcano plot showing the number of DEGs in the day (top figure) and night (bottom figure) in infected A. thaliana plants 14 DAI

**
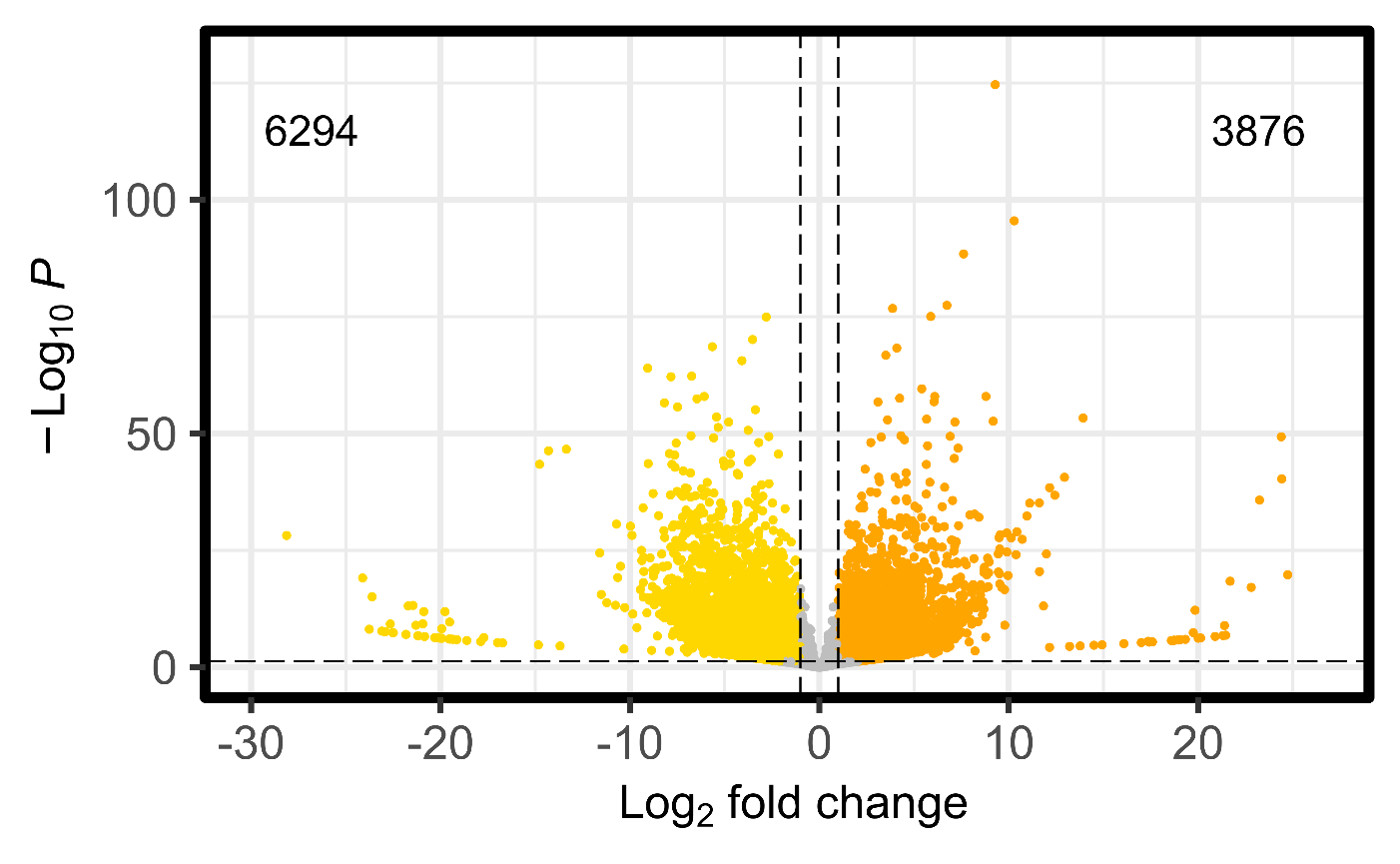

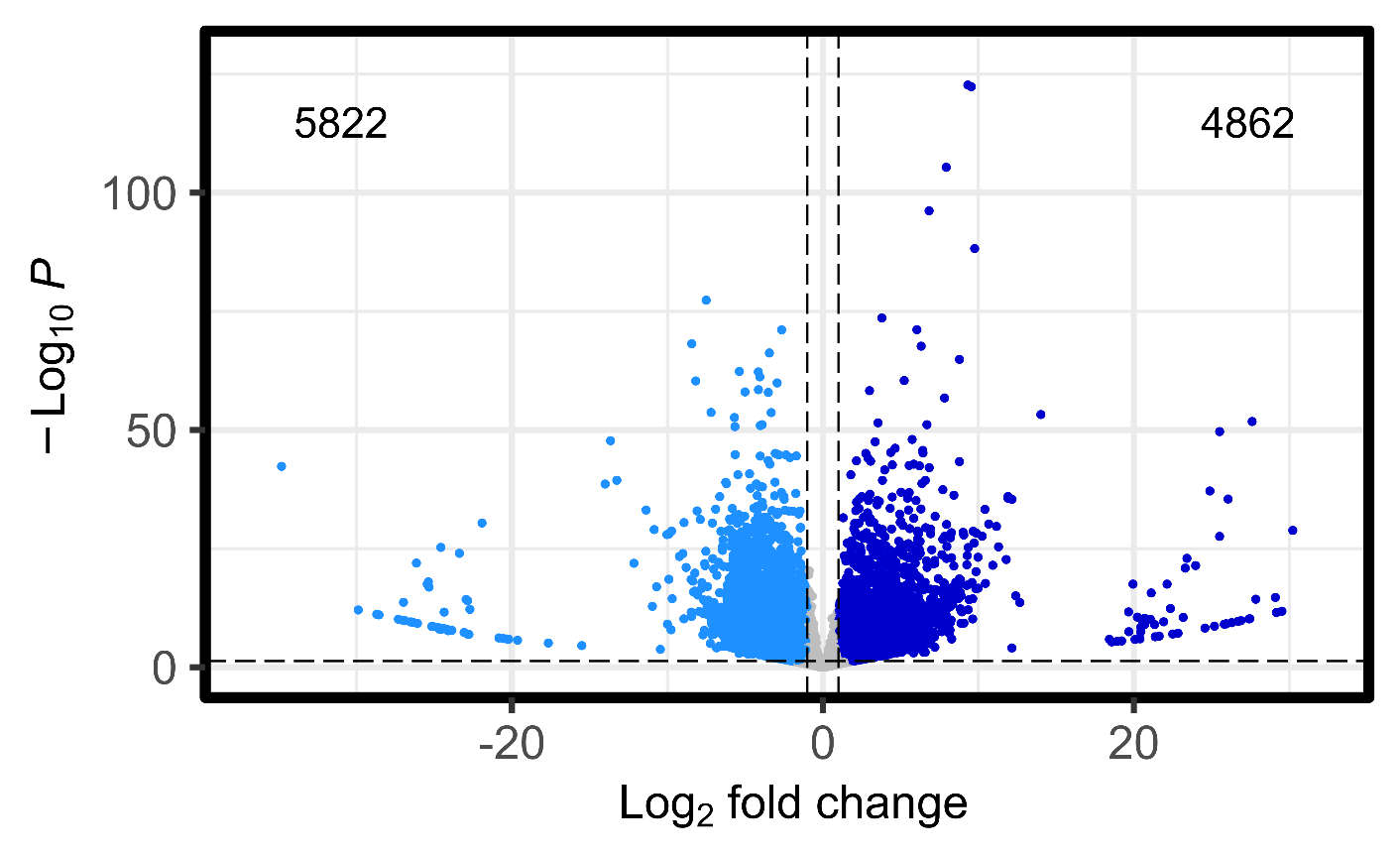
**

Additional File 4 Volcano plot showing the number of DEGs in the day (top figure) and night (bottom figure) in infected A. thaliana plants 21 DAI

Additional File 5 All GO categories enriched in DEGs in A. thaliana roots 14 DAI infected by P. brassicae as in Fig 3. GO categories are organised by decreasing -log10 adjusted p-value (i.e., top to bottom in the relative figure).

| **GO term categories affected by *P.brassicae* infection in *A. thaliana* 14 d.a.i.** | | | | | | | | | |
| --- | --- | --- | --- | --- | --- | --- | --- | --- | --- |
| **Day** | | | | | **Night** | | | | |
| **Upregulated** | | | | | | | | | |
| **ID** | **term** | **count** | **adj_pval** | **zscore** | **ID** | **term** | **count** | **adj_pval** | **zscore** |
| GO:0009636 | response to toxic substance | 55 | 8,92 | 3,64 | GO:0009636 | response to toxic substance | 68 | 11,73 | 3,40 |
| GO:0046906 | tetrapyrrole binding | 89 | 7,47 | 1,38 | GO:0009451 | RNA modification | 101 | 7,86 | 7,06 |
| GO:0048646 | anatomical structure formation involved in morphogenesis | 68 | 5,68 | 1,21 | GO:0140359 | ABC-type transporter activity | 41 | 6,98 | 0,47 |
| GO:0008299 | isoprenoid biosynthetic process | 54 | 4,74 | 0,27 | GO:0090332 | stomatal closure | 26 | 5,69 | 0,39 |
| GO:0048580 | regulation of post-embryonic development | 91 | 4,41 | 0,31 | GO:0000302 | response to reactive oxygen species | 50 | 5,36 | 0,28 |
| GO:0046148 | pigment biosynthetic process | 52 | 4,38 | 1,94 | GO:0002213 | defense response to insect | 32 | 4,97 | 1,06 |
| GO:0038023 | signaling receptor activity | 36 | 4,31 | 0,67 | GO:0048580 | regulation of post-embryonic development | 108 | 4,70 | 0,38 |
| GO:0048046 | apoplast | 70 | 4,22 | 1,20 | GO:0009739 | response to gibberellin | 40 | 4,41 | 0,32 |
| GO:0010287 | plastoglobule | 21 | 3,75 | 2,40 | GO:0048544 | recognition of pollen | 20 | 3,82 | 3,13 |
| GO:0009767 | photosynthetic electron transport chain | 19 | 3,41 | 3,44 | GO:0009991 | response to extracellular stimulus | 79 | 3,60 | 0,11 |
| GO:0042651 | thylakoid membrane | 73 | 3,14 | 5,97 | GO:0045165 | cell fate commitment | 30 | 2,96 | 0,73 |
| GO:0016829 | lyase activity | 65 | 3,06 | 0,12 | GO:0099503 | secretory vesicle | 46 | 2,90 | 0,59 |
| GO:0009739 | response to gibberellin | 31 | 2,94 | 1,26 | GO:0071214 | cellular response to abiotic stimulus | 63 | 2,81 | 0,13 |
| GO:0009644 | response to high light intensity | 19 | 2,87 | 1,15 | GO:0007166 | cell surface receptor signaling pathway | 19 | 2,70 | 0,69 |
| GO:0015267 | channel activity | 41 | 2,86 | 0,47 | GO:2000377 | regulation of reactive oxygen species metabolic process | 21 | 2,63 | 1,53 |
| GO:0102483 | scopolin beta-glucosidase activity | 14 | 2,39 | 0,53 | GO:0009723 | response to ethylene | 60 | 2,45 | 0,26 |
| GO:0010168 | ER body | 7 | 2,35 | 2,65 | GO:1902679 | negative regulation of RNA biosynthetic process | 53 | 2,45 | 0,41 |
| GO:0016054 | organic acid catabolic process | 35 | 2,09 | 0,85 | GO:0008171 | O-methyltransferase activity | 16 | 2,38 | 0,50 |
| GO:2000377 | regulation of reactive oxygen species metabolic process | 17 | 2,02 | 1,21 | GO:0048825 | cotyledon development | 18 | 2,32 | 1,41 |
| GO:0048544 | recognition of pollen | 14 | 1,96 | 0,53 | GO:0000712 | resolution of meiotic recombination intermediates | 7 | 2,21 | 2,65 |
| GO:0000373 | Group II intron splicing | 9 | 1,82 | 0,33 | GO:0042440 | pigment metabolic process | 82 | 2,01 | 0,66 |
| GO:0009630 | gravitropism | 21 | 1,66 | 0,65 | GO:0008283 | cell population proliferation | 27 | 1,90 | 0,96 |
| GO:0008283 | cell population proliferation | 22 | 1,51 | 0,43 | GO:0048235 | pollen sperm cell differentiation | 12 | 1,87 | 0,58 |
| GO:1900865 | chloroplast RNA modification | 6 | 1,45 | 1,63 | GO:0000079 | regulation of cyclin-dependent protein serine/threonine kinase activity | 15 | 1,84 | 0,26 |
| GO:0006638 | neutral lipid metabolic process | 10 | 1,41 | 0,63 | GO:0042793 | plastid transcription | 7 | 1,84 | 1,89 |
|  |  |  |  |  | GO:0002832 | negative regulation of response to biotic stimulus | 15 | 1,78 | 0,77 |
|  |  |  |  |  | GO:0010114 | response to red light | 25 | 1,64 | 0,20 |
|  |  |  |  |  | GO:0010090 | trichome morphogenesis | 19 | 1,63 | 0,23 |
|  |  |  |  |  | GO:0019432 | triglyceride biosynthetic process | 9 | 1,47 | 0,33 |
|  |  |  |  |  | GO:1901681 | sulfur compound binding | 11 | 1,40 | 0,30 |
| **Zero z-score** | | | | | | | | | |
| **ID** | **term** | **count** | **adj_pval** | **zscore** | **ID** | **term** | **count** | **adj_pval** | **zscore** |
| GO:0002238 | response to molecule of fungal origin | 10 | 3,43 | 0,00 | GO:0008299 | isoprenoid biosynthetic process | 74 | 8,81 | 0,00 |
|  |  |  |  |  | GO:0048437 | floral organ development | 72 | 4,62 | 0,00 |
|  |  |  |  |  | GO:1901698 | response to nitrogen compound | 64 | 3,43 | 0,00 |
|  |  |  |  |  | GO:0009608 | response to symbiont | 6 | 1,75 | 0,00 |
|  |  |  |  |  | GO:0009629 | response to gravity | 26 | 1,66 | 0,00 |
| **Downregulated** | | | | | | | | | |
| **ID** | **term** | **count** | **adj_pval** | **zscore** | **ID** | **term** | **count** | **adj_pval** | **zscore** |
| GO:0071456 | cellular response to hypoxia | 86 | 18,78 | -3,23 | GO:0044262 | cellular carbohydrate metabolic process | 144 | 17,31 | -5,33 |
| GO:0044262 | cellular carbohydrate metabolic process | 125 | 17,57 | -4,74 | GO:0003953 | NAD+ nucleosidase activity | 27 | 16,14 | -4,81 |
| GO:0044550 | secondary metabolite biosynthetic process | 107 | 14,08 | -3,77 | GO:0009698 | phenylpropanoid metabolic process | 64 | 14,42 | -3,25 |
| GO:0003953 | NAD+ nucleosidase activity | 24 | 14,04 | -3,27 | GO:0036294 | cellular response to decreased oxygen levels | 87 | 13,71 | -3,11 |
| GO:0009751 | response to salicylic acid | 107 | 11,64 | -4,54 | GO:0016798 | hydrolase activity acting on glycosyl bonds | 127 | 11,94 | -5,06 |
| GO:0012501 | programmed cell death | 59 | 9,99 | -2,21 | GO:0020037 | heme binding | 104 | 9,74 | -0,98 |
| GO:0048584 | positive regulation of response to stimulus | 72 | 7,78 | -1,89 | GO:0009751 | response to salicylic acid | 117 | 9,70 | -2,68 |
| GO:0042537 | benzene-containing compound metabolic process | 29 | 7,18 | -2,79 | GO:0045229 | external encapsulating structure organization | 107 | 9,36 | -3,96 |
| GO:0042802 | identical protein binding | 89 | 6,82 | -0,74 | GO:0016143 | S-glycoside metabolic process | 82 | 9,34 | -1,77 |
| GO:0048437 | floral organ development | 69 | 6,71 | -0,12 | GO:0048646 | anatomical structure formation involved in morphogenesis | 87 | 8,42 | -1,61 |
| GO:0000302 | response to reactive oxygen species | 47 | 6,58 | -0,44 | GO:0048584 | positive regulation of response to stimulus | 84 | 8,32 | -0,44 |
| GO:0062012 | regulation of small molecule metabolic process | 54 | 6,54 | -4,08 | GO:0010087 | phloem or xylem histogenesis | 55 | 7,08 | -4,45 |
| GO:0045229 | external encapsulating structure organization | 81 | 5,53 | -3,22 | GO:2000652 | regulation of secondary cell wall biogenesis | 28 | 6,87 | -4,54 |
| GO:0090332 | stomatal closure | 23 | 5,38 | -1,46 | GO:0042537 | benzene-containing compound metabolic process | 31 | 6,56 | -3,05 |
| GO:1903338 | regulation of cell wall organization or biogenesis | 25 | 5,14 | -4,60 | GO:0015849 | organic acid transport | 46 | 5,92 | -1,18 |
| GO:0009743 | response to carbohydrate | 48 | 4,95 | -0,87 | GO:0010150 | leaf senescence | 83 | 5,86 | -0,77 |
| GO:0016747 | acyltransferase activity transferring groups other than amino-acyl groups | 69 | 4,63 | -0,60 | GO:0010648 | negative regulation of cell communication | 41 | 5,85 | -0,78 |
| GO:0048585 | negative regulation of response to stimulus | 56 | 4,49 | -0,80 | GO:0006811 | monoatomic ion transport | 94 | 5,78 | -0,83 |
| GO:0010087 | phloem or xylem histogenesis | 41 | 4,16 | -3,90 | GO:0015698 | inorganic anion transport | 40 | 5,54 | -1,58 |
| GO:0015698 | inorganic anion transport | 32 | 4,09 | -0,71 | GO:0010143 | cutin biosynthetic process | 16 | 5,41 | -0,50 |
| GO:0009733 | response to auxin | 83 | 3,77 | -0,99 | GO:0008219 | cell death | 68 | 5,30 | -2,43 |
| GO:0010150 | leaf senescence | 64 | 3,75 | -0,75 | GO:0000981 | DNA-binding transcription factor activity RNA polymerase II-specific | 79 | 5,30 | -0,56 |
| GO:0046777 | protein autophosphorylation | 40 | 3,70 | -2,85 | GO:0007623 | circadian rhythm | 49 | 4,99 | -0,43 |
| GO:0042594 | response to starvation | 48 | 3,64 | -0,29 | GO:0090627 | plant epidermal cell differentiation | 62 | 4,92 | -2,29 |
| GO:0140359 | ABC-type transporter activity | 29 | 3,49 | -0,56 | GO:0071365 | cellular response to auxin stimulus | 42 | 4,84 | -0,31 |
| GO:0015849 | organic acid transport | 34 | 3,43 | -0,34 | GO:0042802 | identical protein binding | 94 | 4,33 | -0,62 |
| GO:0006816 | calcium ion transport | 23 | 3,38 | -1,46 | GO:0009735 | response to cytokinin | 39 | 3,62 | -1,12 |
| GO:0048235 | pollen sperm cell differentiation | 13 | 3,02 | -0,28 | GO:0009308 | amine metabolic process | 43 | 3,48 | -2,29 |
| GO:0070417 | cellular response to cold | 13 | 3,02 | -0,28 | GO:0043621 | protein self-association | 35 | 3,32 | -0,51 |
| GO:0043621 | protein self-association | 29 | 2,79 | -0,56 | GO:0034285 | response to disaccharide | 25 | 3,22 | -1,80 |
| GO:0006631 | fatty acid metabolic process | 61 | 2,77 | -0,38 | GO:0019899 | enzyme binding | 81 | 3,01 | -2,33 |
| GO:0009308 | amine metabolic process | 35 | 2,76 | -2,87 | GO:0022603 | regulation of anatomical structure morphogenesis | 50 | 2,84 | -1,70 |
| GO:0009624 | response to nematode | 22 | 2,76 | -1,28 | GO:0046777 | protein autophosphorylation | 43 | 2,83 | -0,15 |
| GO:1901698 | response to nitrogen compound | 52 | 2,72 | -0,28 | GO:0009741 | response to brassinosteroid | 34 | 2,65 | -2,74 |
| GO:0042887 | amide transmembrane transporter activity | 18 | 2,64 | -1,41 | GO:0009705 | plant-type vacuole membrane | 36 | 2,58 | -1,33 |
| GO:0048767 | root hair elongation | 21 | 2,46 | -1,53 | GO:0015295 | solute:proton symporter activity | 13 | 2,57 | -0,83 |
| GO:0009408 | response to heat | 70 | 2,46 | -0,96 | GO:0006880 | intracellular sequestering of iron ion | 8 | 2,47 | -0,71 |
| GO:0019900 | kinase binding | 29 | 2,43 | -3,53 | GO:0009826 | unidimensional cell growth | 96 | 2,45 | -2,04 |
| GO:0010073 | meristem maintenance | 51 | 2,41 | -0,42 | GO:0048046 | apoplast | 72 | 2,44 | -1,18 |
| GO:0009625 | response to insect | 13 | 2,38 | -0,28 | GO:0009624 | response to nematode | 24 | 2,42 | -0,82 |
| GO:0000981 | DNA-binding transcription factor activity RNA polymerase II-specific | 56 | 2,32 | -0,53 | GO:0006833 | water transport | 14 | 2,34 | -1,07 |
| GO:0051644 | plastid localization | 9 | 2,27 | -0,33 | GO:0008236 | serine-type peptidase activity | 43 | 2,33 | -1,98 |
| GO:0005337 | nucleoside transmembrane transporter activity | 11 | 2,24 | -2,11 | GO:0010073 | meristem maintenance | 59 | 2,26 | -0,13 |
| GO:0042578 | phosphoric ester hydrolase activity | 66 | 2,24 | -2,95 | GO:0051119 | sugar transmembrane transporter activity | 19 | 2,18 | -1,61 |
| GO:0071215 | cellular response to abscisic acid stimulus | 59 | 2,19 | -3,78 | GO:0042886 | amide transport | 15 | 2,18 | -1,29 |
| GO:0007389 | pattern specification process | 60 | 2,11 | -1,03 | GO:0010038 | response to metal ion | 51 | 2,05 | -2,66 |
| GO:0009705 | plant-type vacuole membrane | 29 | 2,06 | -1,30 | GO:0008374 | O-acyltransferase activity | 30 | 2,02 | -2,92 |
| GO:0009531 | secondary cell wall | 9 | 2,06 | -3,00 | GO:0009411 | response to UV | 31 | 2,00 | -0,90 |
| GO:0005507 | copper ion binding | 37 | 2,01 | -0,16 | GO:0030570 | pectate lyase activity | 10 | 1,96 | -0,63 |
| GO:0055062 | phosphate ion homeostasis | 9 | 1,91 | -1,00 | GO:0070417 | cellular response to cold | 12 | 1,87 | -0,58 |
| GO:0009269 | response to desiccation | 9 | 1,91 | -1,67 | GO:0035556 | intracellular signal transduction | 90 | 1,87 | -1,26 |
| GO:0009826 | unidimensional cell growth | 77 | 1,82 | -1,71 | GO:0010214 | seed coat development | 17 | 1,87 | -1,70 |
| GO:0004620 | phospholipase activity | 15 | 1,76 | -1,81 | GO:0051049 | regulation of transport | 30 | 1,85 | -1,46 |
| GO:0071214 | cellular response to abiotic stimulus | 48 | 1,64 | -0,29 | GO:0050660 | flavin adenine dinucleotide binding | 37 | 1,84 | -0,82 |
| GO:0009735 | response to cytokinin | 27 | 1,58 | -0,96 | GO:0004551 | dinucleotide phosphatase activity | 6 | 1,84 | -1,63 |
| GO:0008171 | O-methyltransferase activity | 12 | 1,53 | -0,58 | GO:0009615 | response to virus | 25 | 1,83 | -1,00 |
| GO:0030145 | manganese ion binding | 13 | 1,52 | -3,05 | GO:0006631 | fatty acid metabolic process | 65 | 1,67 | -1,86 |
| GO:0050660 | flavin adenine dinucleotide binding | 30 | 1,51 | -0,37 | GO:0009850 | auxin metabolic process | 19 | 1,63 | -0,23 |
| GO:0040034 | regulation of development heterochronic | 13 | 1,47 | -0,28 | GO:0009408 | response to heat | 77 | 1,63 | -1,48 |
| GO:0045926 | negative regulation of growth | 10 | 1,47 | -0,63 | GO:0016829 | lyase activity | 68 | 1,60 | -0,73 |
| GO:0071367 | cellular response to brassinosteroid stimulus | 18 | 1,45 | -2,36 | GO:0051923 | sulfation | 7 | 1,55 | -2,65 |
| GO:0016307 | phosphatidylinositol phosphate kinase activity | 6 | 1,43 | -0,82 | GO:0030145 | manganese ion binding | 15 | 1,54 | -3,36 |
| GO:0015081 | sodium ion transmembrane transporter activity | 11 | 1,32 | -1,51 | GO:0007010 | cytoskeleton organization | 65 | 1,49 | -3,35 |
|  |  |  |  |  | GO:0009269 | response to desiccation | 9 | 1,47 | -1,67 |
|  |  |  |  |  | GO:0090158 | endoplasmic reticulum membrane organization | 6 | 1,42 | -1,63 |
|  |  |  |  |  | GO:0015851 | nucleobase transport | 9 | 1,38 | -1,00 |
|  |  |  |  |  | GO:0009631 | cold acclimation | 15 | 1,31 | -2,32 |

Additional File 6 All GO categories enriched in DEGs in A. thaliana roots 21 DAI infected by P. brassicae as in Fig 3. GO categories are organised by decreasing -log10 adjusted p-value (i.e., top to bottom in the relative figure).

| **GO term categories affected by *P.brassicae* infection in *A. thaliana* 21 d.a.i.** | | | | | | | | | |
| --- | --- | --- | --- | --- | --- | --- | --- | --- | --- |
| **Day** | | | | | **Night** | | | | |
| **Upregulated** | | | | | | | | | |
| **ID** | **term** | **count** | **adj_pval** | **zscore** | **ID** | **term** | **count** | **adj_pval** | **zscore** |
| GO:0015979 | photosynthesis | 123 | 14,82 | 8,75 | GO:0015979 | photosynthesis | 132 | 16,94 | 9,05 |
| GO:0009657 | plastid organization | 121 | 11,02 | 5,36 | GO:0009991 | response to extracellular stimulus | 148 | 14,73 | 1,97 |
| GO:0016054 | organic acid catabolic process | 76 | 8,94 | 0,92 | GO:0009657 | plastid organization | 131 | 12,99 | 6,55 |
| GO:0046148 | pigment biosynthetic process | 91 | 8,43 | 3,25 | GO:0003953 | NAD+ nucleosidase activity | 27 | 11,87 | 0,19 |
| GO:0009646 | response to absence of light | 33 | 8,32 | 0,87 | GO:0000302 | response to reactive oxygen species | 81 | 11,34 | 0,56 |
| GO:0007623 | circadian rhythm | 68 | 6,98 | 0,97 | GO:0042802 | identical protein binding | 151 | 9,49 | 0,08 |
| GO:0009639 | response to red or far red light | 134 | 5,25 | 0,69 | GO:0044282 | small molecule catabolic process | 99 | 9,20 | 0,90 |
| GO:0002213 | defense response to insect | 37 | 3,95 | 0,49 | GO:0009642 | response to light intensity | 143 | 9,17 | 5,44 |
| GO:0005777 | peroxisome | 94 | 2,63 | 0,41 | GO:0015995 | chlorophyll biosynthetic process | 51 | 8,01 | 4,90 |
| GO:0000373 | Group II intron splicing | 13 | 2,17 | 1,39 | GO:0071214 | cellular response to abiotic stimulus | 107 | 7,63 | 1,45 |
| GO:0007166 | cell surface receptor signaling pathway | 21 | 1,81 | 2,40 | GO:0007623 | circadian rhythm | 72 | 7,53 | 2,59 |
| GO:0006641 | triglyceride metabolic process | 14 | 1,79 | 0,53 | GO:0009743 | response to carbohydrate | 81 | 7,52 | 1,00 |
| GO:1901361 | organic cyclic compound catabolic process | 90 | 1,55 | 0,84 | GO:0010150 | leaf senescence | 115 | 6,56 | 1,40 |
| GO:0015749 | monosaccharide transmembrane transport | 12 | 1,49 | 1,15 | GO:0051093 | negative regulation of developmental process | 73 | 5,26 | 0,35 |
| GO:0005315 | inorganic phosphate transmembrane transporter activity | 9 | 1,35 | 1,00 | GO:0071949 | FAD binding | 37 | 4,80 | 1,81 |
|  |  |  |  |  | GO:0043621 | protein self-association | 50 | 4,58 | 0,28 |
|  |  |  |  |  | GO:0015144 | carbohydrate transmembrane transporter activity | 39 | 4,41 | 0,48 |
|  |  |  |  |  | GO:0051253 | negative regulation of RNA metabolic process | 81 | 3,19 | 0,56 |
|  |  |  |  |  | GO:0002213 | defense response to insect | 36 | 3,10 | 0,33 |
|  |  |  |  |  | GO:0008271 | secondary active sulfate transmembrane transporter activity | 9 | 3,00 | 0,33 |
|  |  |  |  |  | GO:0010014 | meristem initiation | 21 | 2,49 | 0,22 |
|  |  |  |  |  | GO:0022411 | cellular component disassembly | 40 | 2,33 | 0,63 |
|  |  |  |  |  | GO:0009704 | de-etiolation | 10 | 2,29 | 0,63 |
|  |  |  |  |  | GO:0040034 | regulation of development heterochronic | 22 | 2,28 | 0,43 |
|  |  |  |  |  | GO:0009739 | response to gibberellin | 45 | 2,28 | 0,45 |
|  |  |  |  |  | GO:0000373 | Group II intron splicing | 13 | 2,01 | 1,39 |
|  |  |  |  |  | GO:1900865 | chloroplast RNA modification | 9 | 1,92 | 2,33 |
|  |  |  |  |  | GO:0010181 | FMN binding | 13 | 1,57 | 0,83 |
|  |  |  |  |  | GO:0045454 | cell redox homeostasis | 16 | 1,48 | 2,50 |
|  |  |  |  |  | GO:0031406 | carboxylic acid binding | 37 | 1,33 | 0,82 |
|  |  |  |  |  | GO:0019432 | triglyceride biosynthetic process | 11 | 1,32 | 0,90 |
| **Zero z-score** | | | | | | | | | |
| **ID** | **term** | **count** | **adj_pval** | **zscore** | **ID** | **term** | **count** | **adj_pval** | **zscore** |
| GO:0080027 | response to herbivore | 18 | 6,49 | 0 | GO:0009636 | response to toxic substance | 94 | 15,27 | 0 |
| GO:1901699 | cellular response to nitrogen compound | 32 | 4,90 | 0 | GO:0010118 | stomatal movement | 78 | 9,44 | 0 |
| GO:0009624 | response to nematode | 36 | 4,40 | 0 |  |  |  |  |  |
| GO:0004033 | aldo-keto reductase (NADP) activity | 12 | 2,49 | 0 |  |  |  |  |  |
| **Downregulated** | | | | | | | | | |
| **ID** | **term** | **count** | **adj_pval** | **zscore** | **ID** | **term** | **count** | **adj_pval** | **zscore** |
| GO:0044262 | cellular carbohydrate metabolic process | 196 | 23,13 | -6,00 | GO:0044262 | cellular carbohydrate metabolic process | 208 | 25,39 | -5,82 |
| GO:0009698 | phenylpropanoid metabolic process | 84 | 18,71 | -4,80 | GO:0044550 | secondary metabolite biosynthetic process | 171 | 17,41 | -8,34 |
| GO:0009636 | response to toxic substance | 94 | 16,84 | -1,24 | GO:0045229 | external encapsulating structure organization | 156 | 13,46 | -7,37 |
| GO:0003953 | NAD+ nucleosidase activity | 29 | 15,42 | -0,93 | GO:0046906 | tetrapyrrole binding | 158 | 12,94 | -1,91 |
| GO:0046906 | tetrapyrrole binding | 158 | 15,42 | -1,75 | GO:0098660 | inorganic ion transmembrane transport | 107 | 12,43 | -1,64 |
| GO:0016143 | S-glycoside metabolic process | 117 | 14,45 | -6,19 | GO:0006721 | terpenoid metabolic process | 120 | 12,11 | -0,55 |
| GO:0036294 | cellular response to decreased oxygen levels | 109 | 14,30 | -3,93 | GO:0036294 | cellular response to decreased oxygen levels | 107 | 11,74 | -3,96 |
| GO:0009991 | response to extracellular stimulus | 139 | 13,31 | -0,93 | GO:0090698 | post-embryonic plant morphogenesis | 99 | 11,65 | -1,51 |
| GO:0045229 | external encapsulating structure organization | 148 | 12,76 | -7,07 | GO:0140359 | ABC-type transporter activity | 60 | 11,13 | -1,29 |
| GO:0009751 | response to salicylic acid | 159 | 12,21 | -5,63 | GO:0009751 | response to salicylic acid | 162 | 11,09 | -5,34 |
| GO:0048046 | apoplast | 126 | 11,47 | -1,07 | GO:0006631 | fatty acid metabolic process | 132 | 11,01 | -1,04 |
| GO:0000302 | response to reactive oxygen species | 76 | 10,23 | -0,46 | GO:1901615 | organic hydroxy compound metabolic process | 158 | 10,82 | -2,23 |
| GO:0098660 | inorganic ion transmembrane transport | 94 | 8,68 | -1,24 | GO:0048046 | apoplast | 127 | 10,03 | -0,44 |
| GO:0010016 | shoot system morphogenesis | 104 | 8,66 | -3,14 | GO:0090627 | plant epidermal cell differentiation | 93 | 7,81 | -4,87 |
| GO:0006721 | terpenoid metabolic process | 106 | 8,62 | -2,33 | GO:0009966 | regulation of signal transduction | 155 | 7,60 | -4,10 |
| GO:0042537 | benzene-containing compound metabolic process | 41 | 8,59 | -2,97 | GO:0042446 | hormone biosynthetic process | 40 | 7,56 | -3,48 |
| GO:0006631 | fatty acid metabolic process | 119 | 8,38 | -1,19 | GO:0006955 | immune response | 114 | 7,50 | -0,75 |
| GO:0090696 | post-embryonic plant organ development | 83 | 7,98 | -1,43 | GO:0015849 | organic acid transport | 63 | 7,27 | -1,64 |
| GO:0016798 | hydrolase activity acting on glycosyl bonds | 150 | 7,89 | -5,88 | GO:0016829 | lyase activity | 123 | 6,77 | -3,70 |
| GO:0090627 | plant epidermal cell differentiation | 89 | 7,64 | -6,04 | GO:0016746 | acyltransferase activity | 127 | 6,66 | -2,40 |
| GO:0006955 | immune response | 110 | 7,60 | -3,05 | GO:0009624 | response to nematode | 42 | 6,55 | -0,31 |
| GO:0016747 | acyltransferase activity transferring groups other than amino-acyl groups | 116 | 7,43 | -2,04 | GO:0009225 | nucleotide-sugar metabolic process | 30 | 6,10 | -3,65 |
| GO:0010817 | regulation of hormone levels | 107 | 7,40 | -5,32 | GO:2000280 | regulation of root development | 39 | 5,74 | -2,08 |
| GO:0015849 | organic acid transport | 61 | 7,34 | -2,94 | GO:0010038 | response to metal ion | 87 | 5,69 | -2,47 |
| GO:0140359 | ABC-type transporter activity | 51 | 7,24 | -1,82 | GO:0010143 | cutin biosynthetic process | 19 | 5,47 | -1,61 |
| GO:0016682 | oxidoreductase activity acting on diphenols and related substances as donors oxygen as acceptor | 21 | 7,08 | -3,71 | GO:0015267 | channel activity | 74 | 5,30 | -1,86 |
| GO:1901615 | organic hydroxy compound metabolic process | 138 | 6,97 | -2,04 | GO:0009741 | response to brassinosteroid | 54 | 5,25 | -2,72 |
| GO:0010118 | stomatal movement | 69 | 6,91 | -1,81 | GO:0009733 | response to auxin | 144 | 5,24 | -2,17 |
| GO:0010087 | phloem or xylem histogenesis | 68 | 6,77 | -5,82 | GO:0048226 | Casparian strip | 13 | 4,87 | -3,61 |
| GO:0042802 | identical protein binding | 134 | 6,56 | -1,21 | GO:0022626 | cytosolic ribosome | 106 | 4,85 | -6,60 |
| GO:0009741 | response to brassinosteroid | 55 | 6,42 | -3,10 | GO:0005856 | cytoskeleton | 124 | 4,80 | -5,75 |
| GO:0048226 | Casparian strip | 14 | 6,39 | -3,74 | GO:0035556 | intracellular signal transduction | 146 | 4,40 | -1,99 |
| GO:0022626 | cytosolic ribosome | 107 | 6,39 | -6,48 | GO:0004620 | phospholipase activity | 29 | 4,35 | -2,41 |
| GO:0010038 | response to metal ion | 85 | 6,15 | -2,71 | GO:0102483 | scopolin beta-glucosidase activity | 24 | 4,31 | -0,41 |
| GO:0048437 | floral organ development | 99 | 5,92 | -2,11 | GO:0010087 | phloem or xylem histogenesis | 63 | 4,25 | -4,66 |
| GO:0010646 | regulation of cell communication | 146 | 5,90 | -5,96 | GO:0007389 | pattern specification process | 112 | 4,23 | -3,59 |
| GO:0016829 | lyase activity | 114 | 5,84 | -3,56 | GO:0009826 | unidimensional cell growth | 148 | 4,20 | -3,45 |
| GO:0009733 | response to auxin | 140 | 5,77 | -3,21 | GO:0008283 | cell population proliferation | 44 | 4,07 | -0,30 |
| GO:0090693 | plant organ senescence | 120 | 5,68 | -1,10 | GO:0048438 | floral whorl development | 77 | 3,89 | -1,03 |
| GO:0010143 | cutin biosynthetic process | 18 | 5,06 | -1,89 | GO:1904680 | peptide transmembrane transporter activity | 21 | 3,83 | -1,09 |
| GO:0015267 | channel activity | 70 | 5,00 | -2,15 | GO:0030029 | actin filament-based process | 49 | 3,81 | -3,00 |
| GO:0009743 | response to carbohydrate | 70 | 4,90 | -0,48 | GO:0051923 | sulfation | 12 | 3,67 | -2,31 |
| GO:0006833 | water transport | 22 | 4,90 | -2,13 | GO:0009615 | response to virus | 40 | 3,63 | -0,95 |
| GO:0019900 | kinase binding | 51 | 4,87 | -4,90 | GO:0090351 | seedling development | 75 | 3,61 | -1,27 |
| GO:0005507 | copper ion binding | 68 | 4,66 | -2,67 | GO:0006972 | hyperosmotic response | 32 | 3,54 | -0,71 |
| GO:0070417 | cellular response to cold | 20 | 4,55 | -1,79 | GO:0006576 | biogenic amine metabolic process | 38 | 3,47 | -3,57 |
| GO:0006972 | hyperosmotic response | 33 | 4,45 | -1,22 | GO:0005509 | calcium ion binding | 85 | 3,36 | -3,80 |
| GO:0051094 | positive regulation of developmental process | 54 | 4,42 | -2,18 | GO:0009408 | response to heat | 122 | 3,23 | -1,99 |
| GO:0009723 | response to ethylene | 89 | 4,37 | -2,23 | GO:0030145 | manganese ion binding | 24 | 3,16 | -2,45 |
| GO:0051093 | negative regulation of developmental process | 67 | 4,33 | -0,61 | GO:0055086 | nucleobase-containing small molecule metabolic process | 124 | 3,10 | -0,54 |
| GO:0008171 | O-methyltransferase activity | 23 | 3,98 | -1,04 | GO:0009629 | response to gravity | 41 | 3,09 | -1,72 |
| GO:0005774 | vacuolar membrane | 69 | 3,92 | -3,49 | GO:0099503 | secretory vesicle | 60 | 3,03 | -2,84 |
| GO:1903338 | regulation of cell wall organization or biogenesis | 31 | 3,86 | -4,49 | GO:0009631 | cold acclimation | 25 | 2,94 | -0,20 |
| GO:0038023 | signaling receptor activity | 50 | 3,68 | -1,13 | GO:0006833 | water transport | 19 | 2,90 | -2,06 |
| GO:0035556 | intracellular signal transduction | 135 | 3,64 | -3,87 | GO:0035266 | meristem growth | 37 | 2,87 | -0,16 |
| GO:1901568 | fatty acid derivative metabolic process | 25 | 3,43 | -1,40 | GO:0016614 | oxidoreductase activity acting on CH-OH group of donors | 69 | 2,82 | -0,12 |
| GO:0090351 | seedling development | 71 | 3,43 | -1,54 | GO:0000981 | DNA-binding transcription factor activity RNA polymerase II-specific | 95 | 2,78 | -0,31 |
| GO:0050660 | flavin adenine dinucleotide binding | 55 | 3,34 | -0,13 | GO:0008171 | O-methyltransferase activity | 21 | 2,73 | -1,09 |
| GO:0008283 | cell population proliferation | 40 | 3,29 | -2,21 | GO:0044273 | sulfur compound catabolic process | 13 | 2,73 | -2,50 |
| GO:0006576 | biogenic amine metabolic process | 36 | 3,28 | -4,00 | GO:0005507 | copper ion binding | 63 | 2,68 | -3,15 |
| GO:0009826 | unidimensional cell growth | 135 | 3,14 | -5,25 | GO:1901698 | response to nitrogen compound | 84 | 2,64 | -0,87 |
| GO:0051923 | sulfation | 11 | 3,13 | -2,71 | GO:0009838 | abscission | 19 | 2,59 | -2,06 |
| GO:0005509 | calcium ion binding | 80 | 3,10 | -4,70 | GO:0032504 | multicellular organism reproduction | 81 | 2,58 | -2,11 |
| GO:0010073 | meristem maintenance | 83 | 3,03 | -2,52 | GO:0008236 | serine-type peptidase activity | 59 | 2,47 | -1,95 |
| GO:0009739 | response to gibberellin | 46 | 3,00 | -0,59 | GO:0042886 | amide transport | 20 | 2,47 | -0,89 |
| GO:0050829 | defense response to Gram-negative bacterium | 13 | 2,94 | -1,94 | GO:0008378 | galactosyltransferase activity | 20 | 2,47 | -2,24 |
| GO:0009735 | response to cytokinin | 47 | 2,83 | -2,19 | GO:0005615 | extracellular space | 46 | 2,38 | -1,77 |
| GO:0000981 | DNA-binding transcription factor activity RNA polymerase II-specific | 91 | 2,81 | -3,04 | GO:0010223 | secondary shoot formation | 17 | 2,30 | -1,21 |
| GO:0042578 | phosphoric ester hydrolase activity | 109 | 2,81 | -3,54 | GO:0000278 | mitotic cell cycle | 85 | 2,16 | -4,23 |
| GO:0008236 | serine-type peptidase activity | 58 | 2,79 | -2,63 | GO:0016311 | dephosphorylation | 70 | 2,10 | -2,63 |
| GO:0008378 | galactosyltransferase activity | 20 | 2,72 | -2,24 | GO:1901607 | alpha-amino acid biosynthetic process | 56 | 2,08 | -1,60 |
| GO:0051253 | negative regulation of RNA metabolic process | 75 | 2,68 | -0,81 | GO:0019199 | transmembrane receptor protein kinase activity | 18 | 2,05 | -0,47 |
| GO:0032504 | multicellular organism reproduction | 78 | 2,68 | -4,08 | GO:0044087 | regulation of cellular component biogenesis | 47 | 2,04 | -3,94 |
| GO:0099503 | secretory vesicle | 56 | 2,63 | -2,94 | GO:0015851 | nucleobase transport | 13 | 2,01 | -0,83 |
| GO:0030029 | actin filament-based process | 43 | 2,62 | -3,81 | GO:0006644 | phospholipid metabolic process | 67 | 1,94 | -1,83 |
| GO:0051302 | regulation of cell division | 30 | 2,42 | -1,10 | GO:0003008 | system process | 11 | 1,87 | -1,51 |
| GO:0009411 | response to UV | 42 | 2,36 | -1,54 | GO:0032879 | regulation of localization | 47 | 1,68 | -1,60 |
| GO:0042886 | amide transport | 19 | 2,33 | -2,52 | GO:0045927 | positive regulation of growth | 17 | 1,51 | -0,24 |
| GO:0030145 | manganese ion binding | 21 | 2,27 | -2,40 | GO:0009555 | pollen development | 104 | 1,43 | -2,16 |
| GO:0000079 | regulation of cyclin-dependent protein serine/threonine kinase activity | 20 | 2,25 | -1,79 |  |  |  |  |  |
| GO:0015294 | solute:monoatomic cation symporter activity | 17 | 2,24 | -0,73 |  |  |  |  |  |
| GO:0043094 | cellular metabolic compound salvage | 29 | 2,20 | -0,56 |  |  |  |  |  |
| GO:0043621 | protein self-association | 40 | 2,16 | -0,63 |  |  |  |  |  |
| GO:0009631 | cold acclimation | 22 | 2,12 | -1,28 |  |  |  |  |  |
| GO:0009408 | response to heat | 109 | 2,08 | -1,25 |  |  |  |  |  |
| GO:0008970 | phospholipase A1 activity | 9 | 2,02 | -3,00 |  |  |  |  |  |
| GO:0003008 | system process | 11 | 2,00 | -1,51 |  |  |  |  |  |
| GO:0000278 | mitotic cell cycle | 80 | 1,96 | -5,81 |  |  |  |  |  |
| GO:0099080 | supramolecular complex | 113 | 1,87 | -4,80 |  |  |  |  |  |
| GO:0005615 | extracellular space | 42 | 1,87 | -3,09 |  |  |  |  |  |
| GO:1901607 | alpha-amino acid biosynthetic process | 52 | 1,77 | -1,11 |  |  |  |  |  |
| GO:0010214 | seed coat development | 20 | 1,44 | -2,24 |  |  |  |  |  |
| GO:0009606 | tropism | 49 | 1,32 | -2,14 |  |  |  |  |  |
| GO:0055086 | nucleobase-containing small molecule metabolic process | 105 | 1,31 | -0,88 |  |  |  |  |  |


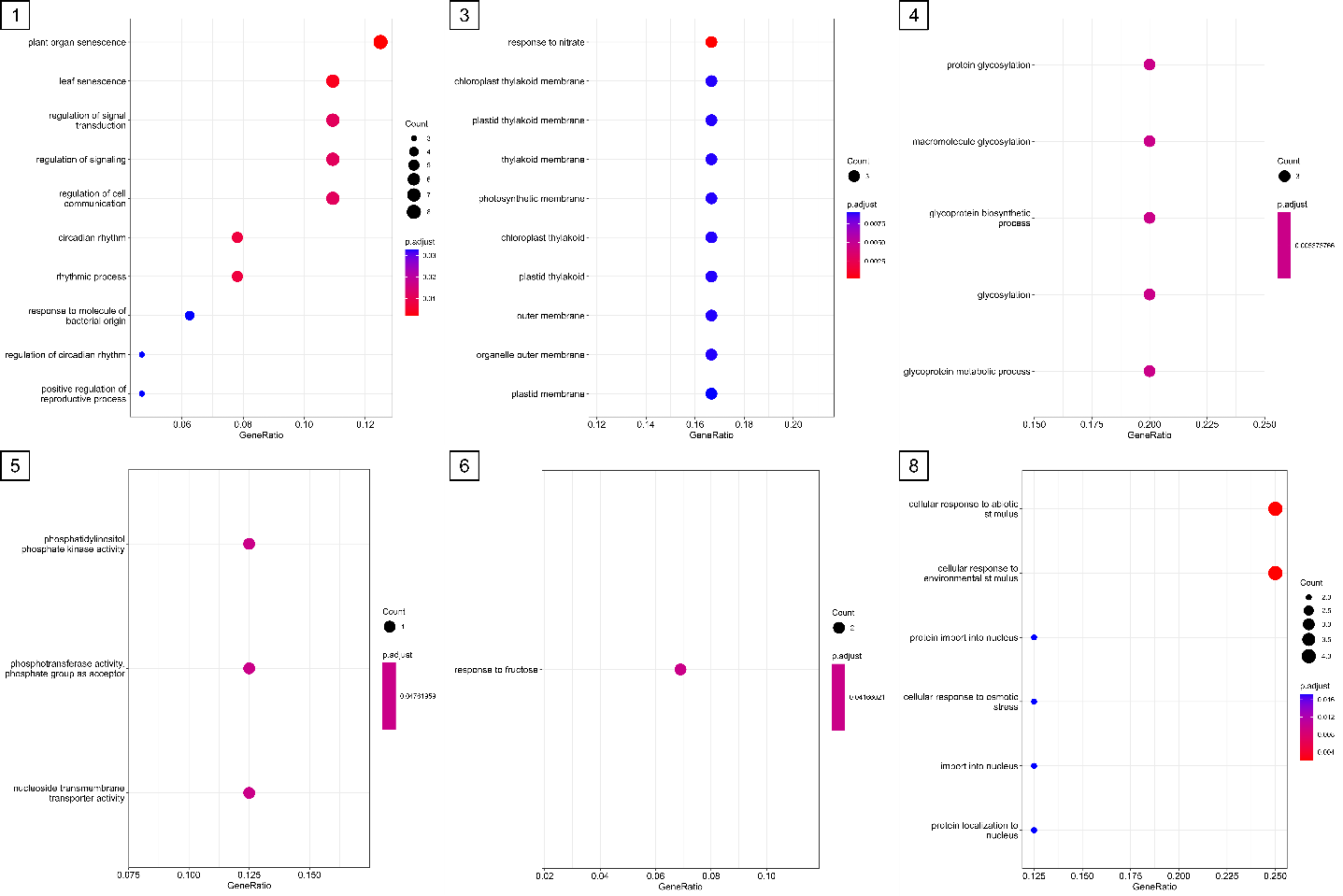


Additional File 7 GO terms overrepresentation analysis of clusters of DEGs driven by the interaction between infection and time of the day 14 DAI


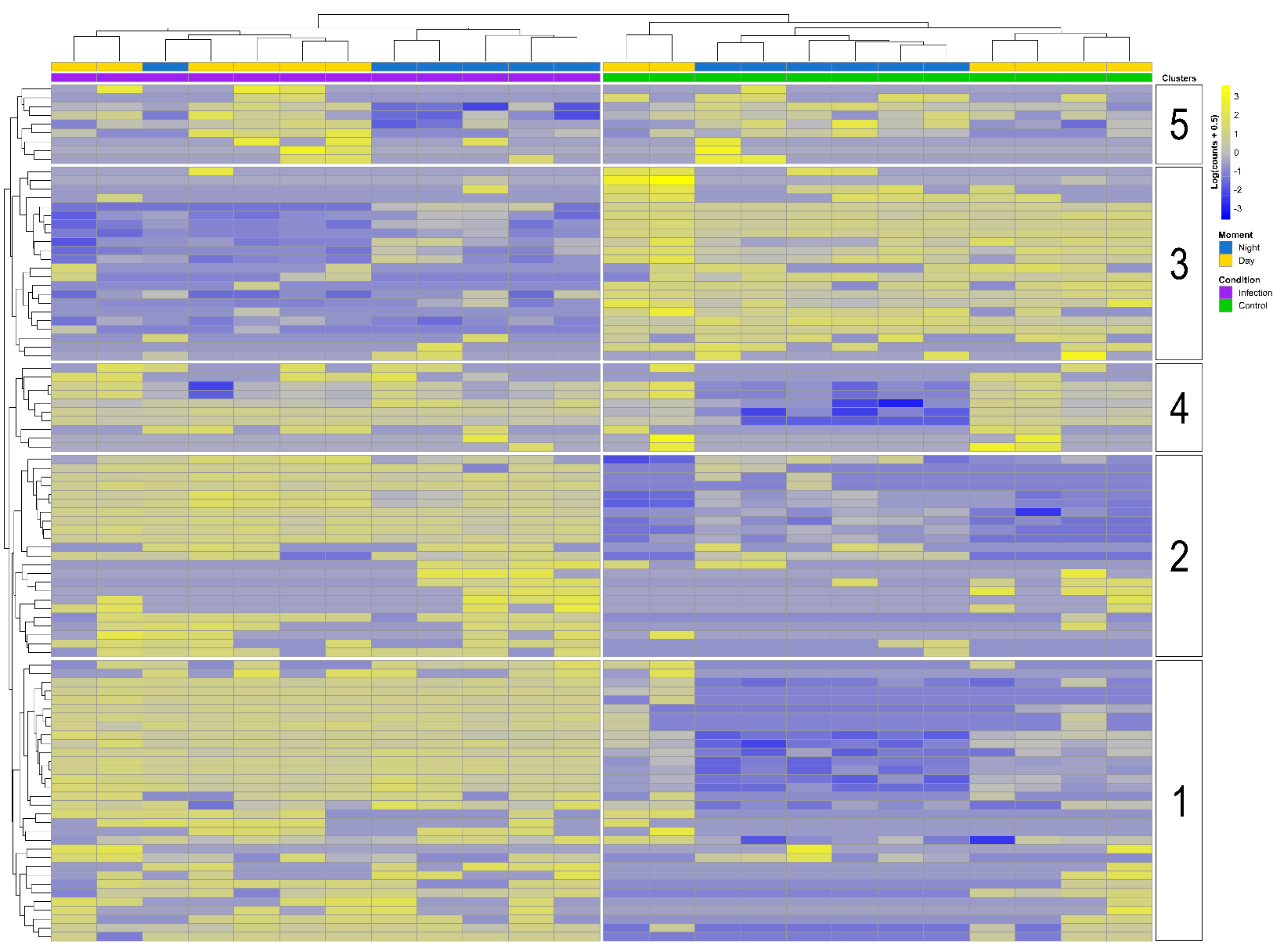


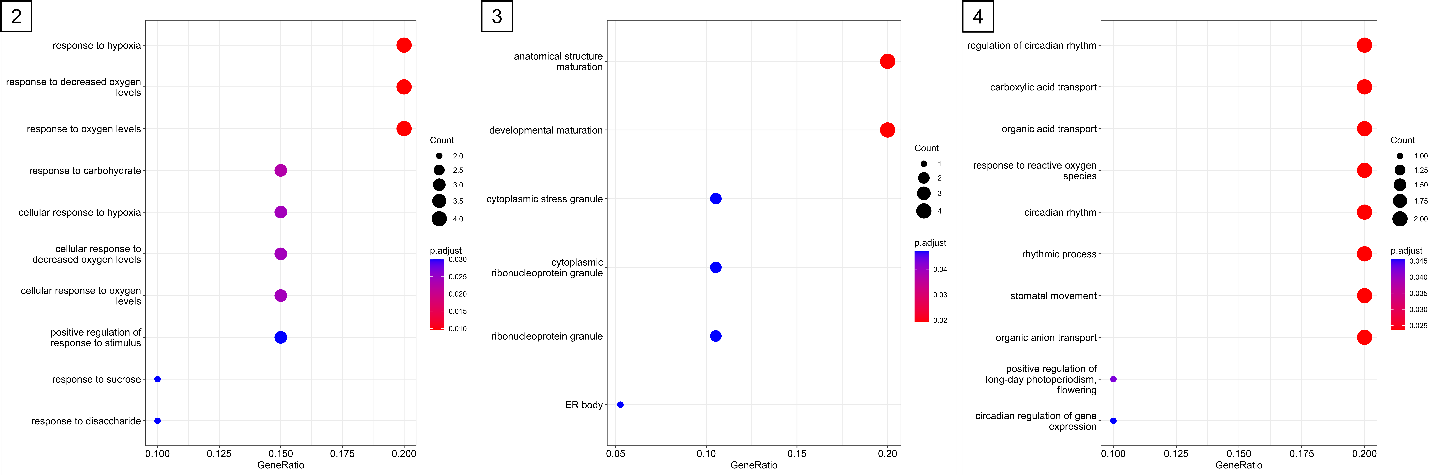


Additional File 8 Heatmap highlighting DEGs explained by the interaction between infection and time of the day as detected in infected A .thaliana roots 21 DAI The row-normalised and log transformed gene counts are shown for each sample separately. The status of each sample is described by a combination of green (control plant) or purple (infected plant) and yellow (day) or (blue) bars. DEGs with similar patterns of expression have been grouped in 5 clusters (numbers in white boxes). GO terms overrepresentation analysis of clusters (2, 3 and 4) of DEGs driven by the interaction between infection and time of the day 21 DAI

<https://fileshare.uibk.ac.at/f/03d6ddfe1e6145bca1a6/?dl=1>

Additional File 9 Enriched GO terms and corresponding genes underpinning figure 2 and 3 before data reduction.
