# Supplementary material for: Under the Cover of Darkness: A Transcriptomic Exploration of Clubroot During the Night
Source: Plant Direct. 2026 Jun 8;10(6):e70176. doi: 10.1002/pld3.70176 (PMC13247141; doi:10.1002/pld3.70176)
Supplement: Supplementary file 1 — Data S1: Peer Review. [file PLD3-10-e70176-s001.pdf]

Plant Direct
Open Access

Under the cover of darkness: a transcriptomic exploration of clubroot during the night

Andrea Garvetto, Susann Auer , Freia Benade , Michaela Hittorf , Jutta Ludwig-Müller , Sigrid Neuhauser

Decision letter Round 1:

December 18, 2025

Andrea Garvetto
Universitat Innsbruck Institut fur Mikrobiologie
Innsbruck 6020
Austria

RE: Under the cover of darkness: a transcriptomic exploration of clubroot during the night.

Dear Dr. Garvetto:

Thank you for submitting to Plant Direct. All required reviews have been returned and we have now finished our evaluation of your manuscript. In light of the reviewers' and editor's comments, further revisions are needed before the paper can be accepted for publication in Plant Direct.

Please view the editors' and reviewers' comments below and use their suggestions as a guide while you work on your revision.

When uploading the revised version of this article, please be sure to include the following:

-A word document that contains your response to the reviewers. You should respond to each reviewer comment and note the changes made to the manuscript. If you do not agree with a reviewer's comment and choose not to make a suggested revision, please explain why. Please try to provide as complete an answer as possible to each reviewer's criticisms in the "response to reviewers" section.
-A tracked changes document
- A clean version of the latest version of the manuscript

Dear Authors

I would also suggest some comments to imrove the Manuscript, apart from the reviewer comment

The Fig 4 and 5 heat maps must be accompanied by some data which shows which signaling pathways are altered and are part of these clusters. Especially the ones with day-night variation. Its very difficult to go back to SI for each information and without this information the analysis does not make any sense. Also since your work in Arabidopsis, can you do a functional test with atleast one gene identified from this huge transcriptome analysis that is novel or could show day-night infection divergences eg. RNA modification, or any common hormone pathway genes identified from cluster and effects on infection process in mutant at dark, which was previously not known

To upload your revision, please click the link below.
https://plantdirect.msubmit.net/cgi-bin/main.plex?el=A3Lr1pI1A5pWp3I3A9ftdOczDjQJybcx6HBN5XFw33AZ

In order to provide as timely a service as possible, we ask that your revision is resubmitted within three months after receipt of this request. If an extension is needed, please send a request, along with a brief explanation, to the editorial office at plantdirect@wiley.com .

Thank you very much for giving us an opportunity to review your work. I look forward to receiving the next version.

Sincerely,
Jyothilakshmi Vadassery

-- Editor comments:
Dear Authors

I would also suggest some comments to imrove the Manuscript, apart from the reviewer comment

The Fig 4 and 5 heat maps must be accompanied by some data which shows which signaling pathways are altered and are part of these clusters. Especially the ones with day-night variation. Its very difficult to go back to SI for each information and without this information the analysis does not make any sense. Also since your work in Arabidopsis, can you do a functional test with atleast one gene identified from this huge transcriptome analysis that is novel or could show day-night infection divergences eg. RNA modification, or any common hormone pathway genes identified from cluster and effects on infection process in mutant at dark, which was previously not known

--- Reviewer comments:
Reviewer #1:

In the current manuscript, the authors present the first-time resolved transcriptome analysis for day and night at intermediate (14 days post-inoculation) and mature (21 days post-inoculation) stages of clubroot disease in Arabidopsis thaliana, providing in-depth details of infection dynamics of P. brassicae in Arabidopsis thaliana . However, I have few comments:
The authors used both Salmon and STAR pipelines here, but generally Salmon is designed to work directly on raw fastq files. What was the rationale for using STAR and Salmon together?
Authors used pooled counts in the DESeq2 pipeline, while DESeq2 requires raw counts per sample. Pooling read counts from multiple time points before differential expression analysis compromises the statistical rigor.
The authors could consider using the MERLIN (Modular regulatory network learning with per gene information) pipeline to make the transcriptome analysis more informative.
Most figures are not very informative and readers may get lost. Fonts could be larger in GO plots and heatmaps.
The authors can limit heatmap to key genes rather than clustering all genes. The discussion of the key genes are also missing. Authors have discussed the GO category in the result which is less informative.
The authors can summarize GO terms using ranked bar plots, dot plots, or network diagrams instead of dense 2d plots.
The manuscript content is vaguely written despite the large transcriptome dataset.
The supplemental figure1 and 2 can be part of the main figure.

Decision Letter Round 2:

My 12, 2026

Andrea Garvetto

Universitat Innsbruck Institut fur Mikrobiologie

Innsbruck 6020

Austria

MSID: 2025-01844-TWR1

MS TITLE: Under the cover of darkness: a transcriptomic exploration of clubroot during the night.

Dear Dr. Garvetto:

I am pleased to inform you that your manuscript "Under the cover of darkness: a transcriptomic exploration of clubroot during the night." has been accepted for publication in Plant Direct.

Your article will appear online in the next available issue of Plant Direct. To ensure your article gets published as quickly as possible, please pay attention to the steps detailed below. We have found that most of the delays happen at this stage, especially at the payment stage, so please respond as quickly as possible when prompted.

License Agreement: Once your article has been accepted it will move to Production and undergo admin and file checking - you may receive an email with any queries we have at this stage. When all required items are received by the publisher and queries resolved, the corresponding author will receive an email from Wiley's Author Services system which will ask them to log on at https://authorservices.wiley.com/bauthor and will present them with the appropriate license for completion. Your article cannot be published until both the signed license agreement and payment of the article fee have been received.

Payment of the Open Access Article Publication Fee: All articles published in Plant Direct are fully open access: immediately and freely available to read, download and share. Plant Direct charges a publication fee to cover the publication costs. The corresponding author for this manuscript should have already received a quote from the payments team (cs-openaccess@wiley.com) with the estimated article publication fee; please email cs-openaccess@wiley.com if this has not been received. The corresponding author should log on to the Wiley Author Services site, where the publication fee can be paid by credit card or an invoice. Pro Forma can also be requested. Payment of the publication charge must be received before the article will be published online.

Proofs: You will have the opportunity to look over your paper once more when you receive the author proofs for your article. The proofs will be with you in approximately two weeks. Please note that, in addition to publishing reviewer comments, the author's responses to review comments will also be published alongside the final version of the paper. If you would not like the author's responses to be published, please contact the editorial office at plantdirect@wiley.com .

Promotion of your article: You can help your research get the attention it deserves! Check out Wiley's free Promotion Guide for best-practice recommendations for promoting your work at www.wileyauthors.com/eeo/guide. And learn more about Wiley Editing Services which offers

professional video, design, and writing services to create shareable video abstracts, infographics, conference posters, lay summaries, and research news stories for your research at www.wileyauthors.com/eeo/promotion.

Thank you again for your contribution to Plant Direct. If you have any questions, feel free to contact the editorial office at plantdirect@wiley.com .

Sincerely,

Jyothilakshmi Vadassery

Jonathan Ingram

Editor, Plant Direct

-- Editor comments

The authors have done the corrections as suggested by reviewer. They could consider an alternate to 2D dense plots. But since there are no real scientific critiques the manuscript may go ahead

-- Reviewer comments:

Reviewer #1:

Thank you for addressing my concern. However, I remain unconvinced as to why the authors present dense 2D plots in Figures 3 and 4 instead of more pinpointed information that would better inform readers. This should be modified.

Response letter Round 1:

Dear editors at Plant Direct,

17 April 2026

We are very thankful for the reviews and suggestions received and for the involvement demonstrated in

enhancing this manuscript.

We strived to amend and improve the manuscript as per the suggestions received and hope that this

new version will be deemed suitable for publication in your journal.

The co-authors and I remain available for further corrections and clarifications.

Kind regards,

Andrea Garvetto, PhD

Postdoctoral researcher

University Innsbruck

Institute of Microbiology

Technikerstraße 25

6020 Innsbruck

Viktor-Franz-Hess Haus, 2nd floor, room 2/14

Tel.: +43 512 507-51241

Mail: andrea.garvetto@uibk.ac.at

Dear Authors

I would also suggest some comments to imrove the Manuscript, apart from the reviewer comment

The Fig 4 and 5 heat maps must be accompanied by some data which shows which signaling pathways

are altered and are part of these clusters. Especially the ones with day-night variation. Its very difficult to

go back to SI for each information and without this information the analysis does not make any sense.

Also since your work in Arabidopsis, can you do a functional test with atleast one gene identified from

this huge transcriptome analysis that is novel or could show day-night infection divergences eg. RNA modification, or any common hormone pathway genes identified from cluster and effects on infection process in mutant at dark, which was previously not known

To upload your revision, please click the link below.

https://plantdirect.msubmit.net/cgi

bin/main.plex?el=A3Lr1pl1A5pWp3I3A9ftdOczDjQJybcx6HBN5XFw33AZ

In order to provide as timely a service as possible, we ask that your revision is resubmitted within three months after receipt of this request. If an extension is needed, please send a request, along with a brief explanation, to the editorial office at plantdirect@wiley.com .

Thank you very much for giving us an opportunity to review your work. I look forward to receiving the next version.

Sincerely,

Jyothilakshmi Vadassery

-- Editor comments:

Dear Authors

I would also suggest some comments to improve the Manuscript, apart from the reviewer comment

The Fig 4 and 5 heat maps must be accompanied by some data which shows which signalling pathways are altered and are part of these clusters. Especially the ones with day-night variation. Its very difficult to go back to SI for each information and without this information the analysis does not make any sense.

We understand the editor's point and would like to use this occasion to better link the discussion and results sections of our paper. Indeed, the discussion already focuses on the disruption of circadian genes that emerged from a closer look into the expression pattern of gene affected by the interaction effect (infection * diel time) at 14 DAI (i.e., the heatmap in Fig.4). Considering the editor's suggestion and taking into account one of the reviewer's comments (see below); we decided to modify Fig. 4 to highlight the influence of clubroot on circadian oscillations. Starting from the same general analysis presented before, we now focus on cluster 1 to identify the disruption of the circadian rhythmicity (GO overrepresentation in cluster 1, Fig. 4 B) and focus on 6 circadian genes unambiguously affected by the infection (Fig. 4 C). We also propose to move Fig. 5 to Additional Information 9 altogether, since the influence of clubroot on diel oscillations in senescing plant is less clear and we feel it does not justify the additional complexity of Fig. 4. After these modifications, we deem the paper now better links the discussion (already interpreting extensively the influence of clubroot on circadian genes) to the result section. We hope this is the type of improvement the editor was hoping for.

Also since your work in Arabidopsis, can you do a functional test with at least one gene identified from this huge transcriptome analysis that is novel or could show day-night infection divergences eg. RNA modification, or any common hormone pathway genes identified from cluster and effects on infection process in mutant at dark, which was previously not known

We appreciate and understand the editor's point and future experimental validation on the results of this explorative paper are indeed planned.

In the meantime, we attempted to further support our results with a RT-qPCR targeting the

most affected genes from our dataset, i.e. pentatricopeptide-repeat containing proteins involved in organellar RNA modification (primers PPRChl and PPRMit, for chloroplast-targeting and mitochondria-targeting PPR-containing proteins) and the core circadian gene PRR7 (see primer table below).

Transcript_id

gene

Primer F

Sequence

Primer R

Sequence

Source

T°

TRINITY_GG_47042

TRINITY_GG_47525

TRINITY_GG_25665

AT4G37380

APRR7

AT5G61030

PPR_Chl_F

APRR7_F

PPR_Mit_F

GAAGAGACAGAGAAAGAGCAA

GTAAGCTCAGCAGGATCTTCAT

GGATCTAGCGGCAACACTTAT

PPR_Chl_R

APRR7_R

PPR_Mit_R

AGCACCAAACCGGGAAG

CTATAACCTTGTCCATGTCGCT

GGAAGTAGTGGTTATGGTAGCG

This study

This study

This study

56.8

58.3

58.6

AT1G50010

TUBA2

ATUA2_F

TGCTTTCGTTCACTGGTATGT

ATUA2_R

AGCACCAACCTCTTCGTAATC

This study

59.0

The primers were designed based specifically on our dataset in order to target conserved regions present in all isoforms detected in our experiment. Unfortunately, our attempt has not gathered positive results as of yet; as primer efficiency still needs to be improved. We hope that the editor appreciates that the sheer production of the biological material for our experiment takes ~ 3 months (in ideal conditions). We deem that repeating the entire experiment would be too much work for this first explorative paper, which we intended as a springboard for future more targeted studies. -- Reviewer comments:

Reviewer #1:

In the current manuscript, the authors present the first-time resolved transcriptome analysis for day and night at intermediate (14 days post-inoculation) and mature (21 days post-inoculation) stages of clubroot disease in Arabidopsis thaliana, providing in-depth details of infection dynamics of P. brassicae in Arabidopsis thaliana. However, I have few comments:

The authors used both Salmon and STAR pipelines here, but generally Salmon is designed to work directly on raw fastq files. What was the rationale for using STAR and Salmon together?

We thank the reviewer for spotting this mistake. The pre-aligning with STAR was performed as part of a parallel strategy on the same dataset using featureCounts for gene-level quantification that is not presented in this study. We are therefore very thankful for highlighting this genuine mistake!

We corrected the statement as follows (ll.472-474):

“Expression estimates per transcript were obtained from raw reads with Salmon (Patro et al., 2017).”

Authors used pooled counts in the DESeq2 pipeline, while DESeq2 requires raw counts per sample. Pooling read counts from multiple time points before differential expression analysis compromises the statistical rigor.

We would like to thank the reviewer for this comment. This is a phrasing issue and we are thankful to the reviewer for raising this point. The DESeq2 pipeline was run on the salmon quantification outputs sample-wise, without any summation (pooling) of the reads. The contrast set in DESeq2 simply took in to account the assignment of each sample to day and night for comparison and computation of DEGs. We modified the statement as follows to clarify (ll. 477-484):

“DESeq2 Bioconductor package (Love et al., 2014) in R (R Core Team, 2021) was used to estimate differential gene expression. In order to detect differences in the response of plants to infection by P. brassicae separately during the day and during the night, differentially expressed genes (DEGs) were calculated by comparing all samples from day time points against all samples from night time points. Briefly, samples from time points 5 hr 50’ and 10 hr 40’ after artificial dawn (i.e., belonging to the day) were compared to samples from time points 15 hr 30’ and 19 hr 20’ after artificial dawn (i.e., belonging to the night).”

The authors could consider using the MERLIN (Modular regulatory network learning with per gene

information) pipeline to make the transcriptome analysis more informative.

We are thankful to the reviewer for this suggestion. We will certainly consider MERLIN to analyse regulatory networks among the downstream analyses we plan on this dataset.

Most figures are not very informative and readers may get lost. Fonts could be larger in GO plots and heatmaps.

We have improved the font in the GO plots in order to make it more readable. Unfortunately, we cannot increase the font more without generating overlaps which will hinder the interpretability of the matching bubbles. Other figures have been amended in order to make them more informative and better linked to our discussion (detailed below).

The authors can limit heatmap to key genes rather than clustering all genes. The discussion of the key genes are also missing. Authors have discussed the GO category in the result which is less informative.

We agree that the key circadian genes affected by the interaction between diel time and infection are not well-highlighted in the results section and in the figures (particularly the heatmaps, as highlighted by the editor as well); although they are extensively examined in the discussion section (ll. 345-409). To improve this, we propose a new figure helping to re-focus the general heatmap on the six genes whose rhythmicity has been found to be affected by infection at 14 DAI. The new composite figure now contains the GO term overrepresentation dotplot of cluster 1 (from Additional File 7) and a smaller heatmap focussing on the 6 genes discussed above. To further streamline the paper, we propose to move the less informative heatmap of the interaction between time and infection at 21 DAI to the Additional File 8. Coincidentally, we amended the Results section and added a new paragraph (ll. 206 219):

"Given the relevance of temporal dynamics in our investigation, genes associated to GO terms "Circadian rhythm" (GO:0007623), "Rhythmic process" (GO:0048511) and "Regulation of circadian rhythm" (GO:0042752) are specifically selected to further clarify the impact of P. brassicae infection on their diel rhythmicity (Fig. 5 B). The six genes are either core components of the plant circadian oscillator, such as GIGANTEA (GI), PSEUDO-RESPONSE REGULATOR 3 (PRR3), 7 (PRR7) and 9 (PRR9); or genes known to be under strong circadian regulation such as PHYTOCHROME INTERACTING FACTOR 4 (PIF4) and NIGHT LIGHT-INDUCIBLE AND CLOCK-REGULATED 1 (LNK1). Consistently with their identification, their expression pattern shows diurnal activation and nocturnal suppression in control conditions, whilst a continuous expression regardless of the time of the day can be observed during clubroot infection (Fig. 5 C). Overall, fewer genes were impacted by the interaction between time of day and infection at 21 days post-infection (DAI) compared to 14 DAI (96 genes with functional annotations). The clustering of the samples cannot discriminate day and night but it maintains a clear separation along infected and control plants, in agreement with the already identified decrease of the temporal component in driving gene expression in older plants (Additional File 8)."

Lines 220-229 have been removed as they expanded on the former main Fig. 5, now Additional file 8. We hope this helps better highlighting the impact of infection on rhythmic processes and increasing the readability by better linking the Results and Discussion sections. No further amendments were deemed necessary in the corresponding part of the Discussion section apart from appropriate reference to the new Fig. 5 and Additional File 8 (ll. 349, 358). We take this occasion to highlight the removal of a

mistaken mention of LNK4 as one of the genes whose rhythmicity is affected by the infection at ll. 358, 361 and 362.

The authors can summarize GO terms using ranked bar plots, dot plots, or network diagrams instead of dense 2d plots.

Although we agree with the reviewer that the GO enrichment figures are dense of information, we would like to point out that these figures have been chosen because of their ability to show information about the overall expression level within each GO category and provide a complete snapshot of the expressional landscape, instead of separating the genes into a priori upregulated and downregulated categories regardless of their association with a given biological process. We therefore prefer to display the data in this format.

The manuscript content is vaguely written despite the large transcriptome dataset.

Considering that this paper is the first exploration of a substantial dataset, we think that a certain degree of vagueness is justifiable. Nevertheless, we strived to discuss specific processes and important genes involved in them specifically to avoid being superficial. We hope that amending the results and strengthening the link between the results and the discussion (as per the above corrections) will help reducing the perceived vagueness of the paper.

The supplemental figure1 and 2 can be part of the main figure.

We agree with the reviewer. The former Additional File 1 has been slightly modified to better fit the page and moved to the main document. The caption now reads: “Figure 1 Phenotypical traits of rosette (left) and root (right) samples for specimen of healthy (A, B, E and F) and infected (C, D, G and H) A. thaliana Col-0 harvested at 14 DAI and 21 DAI. Scale bars = 5 mm.”
